# Experimental Validation of the Direct Kinematics of a Passive Stewart-Gough Platform with Modified Cardan Joints Using Integrated Absolute Linear Encoders

**DOI:** 10.3390/s26030771

**Published:** 2026-01-23

**Authors:** Martin Bem, Aleš Ude, Bojan Nemec

**Affiliations:** 1 Department of Automatics, Biocybernetics, and Robotics, Jožef Stefan Institute, 1000 Ljubljana, Slovenia; ales.ude@ijs.si (A.U.); bojan.nemec@ijs.si (B.N.); 2 Faculty of Electrical Engineering, University of Ljubljana, 1000 Ljubljana, Slovenia

**Keywords:** Stewart-Gough platform, parallel robots, kinematics, Cardan joints, reconfigurable fixtures

## Abstract

This paper presents the experimental validation of a computational kinematic model for a passive Stewart–Gough platform equipped with modified Cardan joints. The introduced joint geometry significantly increases structural stiffness but invalidates the standard spherical joint assumption commonly used in hexapod kinematic formulations. To address this, we develop an efficient numerical optimization-based framework that solves both the direct and inverse kinematics without relying on simplified joint models. Furthermore, to enable autonomous and absolute pose measurement, each cylindrical leg joint of the platform is equipped with a LinACE™ absolute linear encoder. This sensor integration transforms the passive mechanism into an IoT-enabled reconfigurable fixture capable of internally sensing, tracking, and recalling its own configuration. The direct kinematics are computed iteratively using a homogeneous transformation formulation and benchmarked against analytical models derived for ideal spherical joints. Experimental results demonstrate sub-millimeter accuracy in pose estimation, confirming the validity of the proposed kinematic model and highlighting the suitability of the sensor-equipped hexapod for industrial flexible fixturing applications.

## 1. Introduction

In today’s highly competitive industrial environment, manufacturers face increasing pressure to achieve high productivity while shortening time-to-market. As product portfolios expand and product life cycles shorten, conventional manufacturing strategies, typically optimized for producing large quantities of a few product types, are no longer sufficient. Modern production systems therefore require a high degree of flexibility and reconfigurability to efficiently handle product diversity and frequent design modifications [1,2].

This need for adaptability has driven advances in flexible robotic systems and reconfigurable fixturing technologies [3]. In this context, we investigate the use of passive Stewart–Gough platforms (hexapods) as modular building blocks for flexible fixtures in low-volume production environments [4,5].

Active parallel mechanisms based on the Stewart–Gough architecture have been widely studied as generic six-degree-of-freedom motion generators in robotics since the pioneering works of Gough and Stewart [6,7]. These architectures are particularly attractive in applications requiring accurate six-degree-of-freedom pose control, for example in motion simulation, flight simulators, and precision positioning stages. A comprehensive overview of the kinematics, dynamics, singularities, and applications of Stewart platforms is provided in the review by Dasgupta and Mruthyunjaya [8].

Industrial implementations of Stewart platforms driven by electromechanical actuators and servo controllers have been reported for motion control and trajectory tracking tasks, such as reproducing cycloidal and ocean wave trajectories [9] and for evaluating commercial motion controllers for hexapod operation [10]. Parallel platforms of this type have also been explored for aerospace-related applications [11] and for base motion compensation in vibration-sensitive systems [12].

In contrast to the active Stewart–Gough platforms discussed above, the hexapods considered in this work are passive mechanisms without dedicated actuators. They are instead equipped with mechanical brakes that lock the structure in a desired configuration (see Section 2). This passivity is advantageous in robotic manufacturing settings: actuation and sensing are already provided by readily available industrial robots that can reposition the hexapod’s top plate once the brakes are released. As a result, the fixture can be realized at substantially lower cost and with reduced integration effort compared to a fully actuated hexapod, while still enabling reconfigurability when needed. To improve static performance and load-bearing capacity, custom-designed Cardan joints have been integrated into the leg assemblies. However, while this design increases rigidity, it also adds complexity to the direct and inverse kinematic analyses.

Accurate geometric modeling and calibration of Stewart–Gough platforms remain active research topics. Early work by Zhuang and Roth introduced leg-wise calibration methods for these mechanisms [13], while a recent survey by Karmakar and Turner provides an extensive review of modern calibration strategies [14]. Reliable kinematic modeling is essential for estimating the pose of each hexapod and ensuring robust production planning, particularly when multiple platforms simultaneously secure a single workpiece. Earlier hexapod prototypes [2] omitted joint encoders to reduce cost. However, in the current design, each leg incorporates a linear encoder, allowing precise measurement of leg extension and thereby improving configuration estimation. Nonetheless, encoder data are only useful in combination with a corresponding accurate kinematic model.

While non-ideal joint effects in Stewart–Gough platforms have been studied previously, most existing approaches either assume simplified analytical joint models with intersecting axes or rely on calibration-based compensation strategies. To the best of our knowledge, no prior work has presented a general numerical formulation that explicitly incorporates non-intersecting, preloaded Cardan joint geometry into the full kinematic constraint equations while remaining applicable to both direct and inverse kinematics.

Using preloaded axis-offset Cardan joints makes standard Stewart–Gough kinematic formulations unsuitable. Previous studies, such as [15], have examined the kinematics and singularities of mechanisms with axis-offset Cardan joints, focusing mainly on identifying all possible solutions. Building on these foundations, this paper presents a new iterative kinematic method for computing a hexapod configuration in the vicinity of a known initial pose. The approach can also be generalized to compute direct and inverse kinematic solutions for hexapods with arbitrary leg geometries.

The main contributions of this paper are as follows: (a) the development and experimental evaluation of a sensorized passive Stewart–Gough platform equipped with preloaded Cardan joints, which significantly reduce structural backlash and improve stiffness; (b) the formulation and optimization of a Cardan-joint-aware kinematic model that explicitly accounts for the true joint geometry and outperforms conventional Stewart–Gough formulations for the proposed mechanism; and (c) a systematic experimental evaluation of pose accuracy, convergence behavior, and numerical stability over a large dataset of hexapod configurations.

The remainder of this paper is organized as follows: Section 2 describes the design and mechanical characteristics of the developed passive hexapod fixtures. Section 3 and Section 4 introduce the proposed iterative kinematic modeling approach and outline its mathematical formulation. Section 5 presents experimental validation and discusses the accuracy and performance of the proposed method. Finally, Section 6 summarizes the main findings and outlines directions for future research.

## 2. Hexapod Design Overview

The mechanical design of the proposed hexapod is based on a passive Gough–Stewart platform engineered to deliver high rigidity and full reconfigurability for robotic assembly applications. The structure comprises two rigid plates: a base plate fixed to the robotic workcell and a top plate outfitted with modular workpiece locating assemblies. These plates are interconnected by six extendable legs, each equipped with a cylindrical joint, an integrated hydro-mechanical brake, and two custom-designed Cardan joints at both ends [16]. This configuration enables the top plate to move freely across all six degrees of freedom during reconfiguration and to be locked precisely in place for operation. When the hydraulic brakes are disengaged, the platform can be repositioned manually or by a robot through a tool-exchange interface mounted on the top plate. Once the desired pose is achieved, the brakes are activated, fixing the structure with exceptional stiffness and positional accuracy.

A key feature of the design is the hydro-mechanical braking system integrated into each leg [17]. Each brake consists of a deformable sleeve surrounding the moving rod and three concentric pressure chambers radially arranged around the outside of the sleeve. In the unpressurized state, the sleeve geometry provides a high radial preload against the rod, generating a large normal force that rigidly locks the leg. When hydraulic pressure is applied to the chambers, the sleeve deforms, releasing the contact pressure and letting the rod slide freely. This pressure-to-release principle means that any loss of hydraulic pressure during operation leads to an inherently safe state in which all legs remain locked. The brake, in its locked state, can withstand substantial axial loads without slippage, and its compact geometry and simple hydraulic interface make it well suited for integration into industrial hexapod fixtures and other reconfigurable support systems. The Cardan joints used in the system introduce several improvements over conventional universal joint configurations. Each joint incorporates non-intersecting rotational axes together with a central hub featuring adjustable preload that eliminates play and micro gaps, which are common sources of backlash.

The separation of axes and the adjustable preload contribute to increased structural stiffness when the brakes are engaged. Additionally, the joint geometry promotes more uniform load distribution, reducing wear on the contact surfaces and improving long-term reliability. Despite these enhancements, the design maintains a compact form factor while providing high load-bearing capacity.

Together, the hydro-mechanical braking system and enhanced Cardan joints enable the hexapod to achieve sub-millimeter repeatability, minimal backlash, and exceptional rigidity while maintaining a fully passive, low-cost design. The modular top plate can be fitted with interchangeable fixtures, such as centering pins and pneumatic lever clamps, allowing adaptation to a wide range of components, for example, various automotive light housings. Furthermore, multiple hexapods can be combined to form a flexible, robotically reconfigurable fixture system. In general, the integration of precision mechanical locking, adjustable kinematic joints, and modular adaptability delivers an optimal balance between flexibility, structural performance, and precision, establishing the hexapod as a highly capable platform for reconfigurable robotic manufacturing systems.

### Encoder Integration

Several authors have employed external vision systems for kinematic calibration of Stewart platforms, including omnidirectional cameras [18] and stereo vision [19]. A broader survey of calibration methods for spatial parallel mechanisms using internal and external sensing is provided by Majarena et al. [20]. Additional approaches exploiting supplementary sensing, such as dedicated motion controllers or industrial servo hardware, have also been reported for calibration and state estimation [9,10].

**Figure 1:**
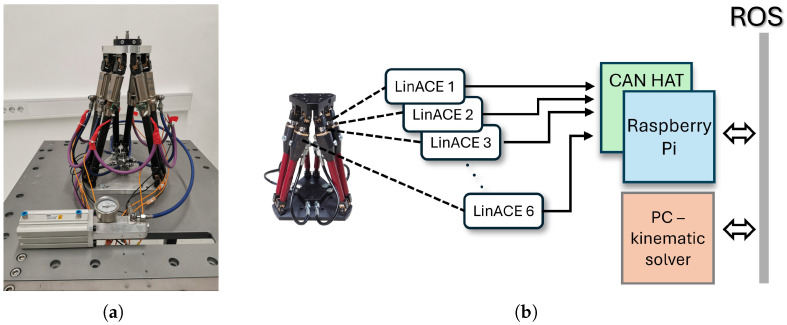
(**a**) Hexapod equipped with LinACE™ absolute linear encoders. (**b**) Schematic overview of the hexapod sensory system.

In contrast to these external techniques, the sensor-integrated hexapod developed here uses LinACE™ absolute linear encoders installed on all six legs (Figure 1a). LinACE technology employs variable magnetic permeability (VMP) sensing: an absolute pseudo-random binary sequence is embedded directly into the steel shaft as regions of differing magnetic permeability, which modulate an applied magnetic field. A compact single-die Hall sensor array reads these field variations, and internal signal processing algorithms reconstruct the absolute position with micrometer-scale accuracy. This solid-state magnetic encoding provides drift-free measurement and preserves absolute position even after power loss.

Each encoder communicates via a CAN bus with a Raspberry Pi Model B running Raspberry Pi OS (64-bit, Kernel 6.1), located in the hexapod base. Because the Raspberry Pi lacks native CAN support, a two-channel isolated MCP2515 CAN controller (Microchip Technology Inc., Chandler, AZ, USA) combined with an SN65HVD230 CAN transceiver (Texas Instruments, Dallas, TX, USA) was used in a CAN-HAT configuration to ensure robust communication. The Raspberry Pi runs a ROS node Robot Operating System [21] that continuously acquires the encoder values and publishes the raw joint data on the ROS network at a rate of 1 Hz. Importantly, the Raspberry Pi functions solely as an edge-level data acquisition device: the inverse kinematic computation of the hexapod pose is performed on an external PC. Once the PC calculates the updated platform pose, the Raspberry Pi records this information in its non-volatile memory (SD card), enabling seamless pose recovery after shutdown or extended storage. The block scheme of the setup is given in Figure 1b.

Integrating absolute encoders significantly increases fixture reliability and operational continuity. With measurement accuracy exceeding that of most industrial robots, robot–fixture alignment becomes simpler and more repeatable. Native ROS communication further supports effortless integration into modular robotic workcells, enabling real-time monitoring and automated calibration routines. Absolute encoders are essential whenever the hexapod is repositioned manually, which frequently occurs in multi-hexapod fixturing scenarios where final alignment must be tuned by a human operator. Without internal sensing, such manual repositioning would render the fixture state unobservable to the robot controller, making autonomous recovery impossible after brake release, servicing, or safety-related shutdowns.

A remaining limitation is that the encoders measure only linear leg extensions rather than the full six-degree-of-freedom pose of the top plate. Accurate pose recovery therefore requires solving the hexapod’s inverse kinematics. Conventional models are not directly applicable due to the use of preloaded Cardan joints with non-intersecting rotational axes, which alter the mechanism’s geometry and constraints. To address this, a dedicated computational method was developed to reliably reconstruct the platform pose despite the non-standard joint configuration. The resulting system combines precise sensing, robust embedded communication, and advanced external computation, delivering a reliable and reconfigurable fixture well suited for modern robotic manufacturing environments.

## 3. Direct and Inverse Kinematics of Parallel Mechanisms

In this section, we summarize the standard Stewart–Gough model used for platforms with spherical joints, which will later serve as a reference when experimentally comparing it to the modified kinematic model developed for our mechanism with preloaded Cardan joints.

**Figure 2:**
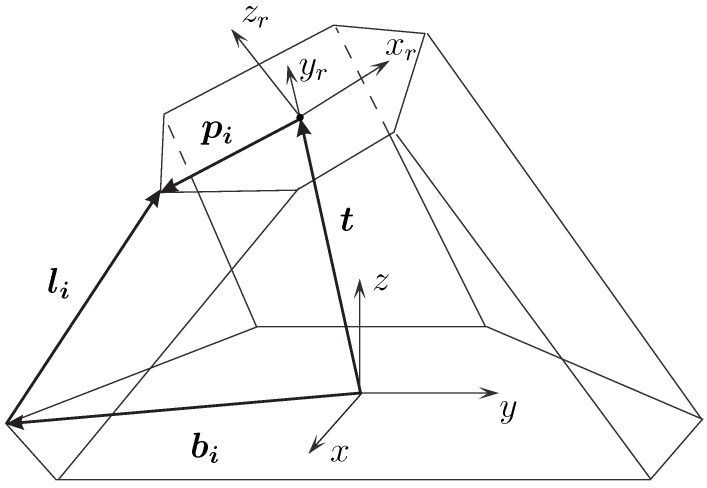
Schematic representation of a Stewart–Gough platform.

For standard Stewart–Gough platforms, the inverse kinematics problem is straightforward [22]. Given the pose of the moving platform, the leg directions follow from the vector loop equation

(1) $$l_{i}=G_{i}\left(x\right)=-b_{i}+t+Rp_{i}\;i=1,\ldots ,6$$

where R is the rotation matrix of the platform, $t={\left[x,y,z\right]}^{T}$ is the position, and $b_{i}$, $p_{i}$ are the fixed leg attachment-point vectors on the base and platform (see Figure 2). The leg lengths follow from

(2) $$l_{i}=∥l_{i}∥.$$

In contrast, the direct kinematics problem (computing x from measured leg lengths) is considerably more difficult and may admit multiple solutions [15]. For obtaining the configuration nearest to a known state, a Newton–Raphson [23] iteration may be used:

(3) $$x_{j+1}=x_{j}-J^{-1}\;\left(G\left(x_{j}\right)-l^{m}\right),$$

where $l^{m}={\left[l_{1}^{m},\ldots ,l_{6}^{m}\right]}^{T}$ are the measured leg lengths and J is the Jacobian

(4) $$J=\frac{\partial G(x)}{\partial x}\in R^{6\times 6}.$$

However, this formulation assumes ideal spherical joints and therefore cannot be applied to our platform, which incorporates twelve preloaded Cardan joints. In this case, the leg direction vectors in (1) are insufficient because the rotational axes of the joints are offset and do not intersect. The kinematics must instead be expressed through homogeneous transformations, as described next.

## 4. Kinematics of a Stewart–Gough Platform with Preloaded Cardan Joints

**Figure 3:**
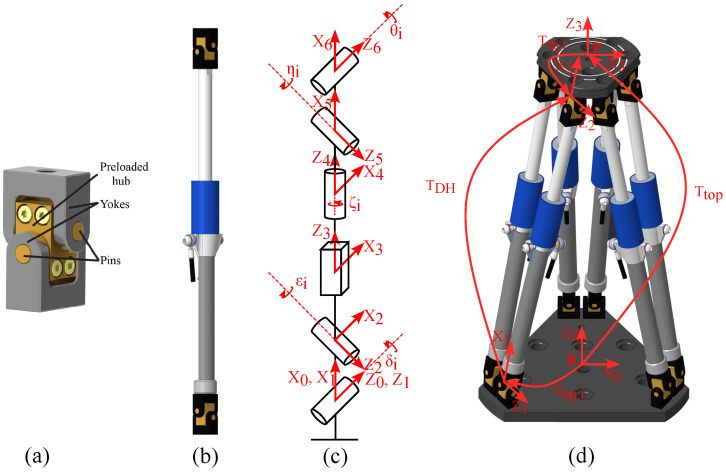
(**a**) Preloaded Cardan joint illustrating offset, non-intersecting axes; (**b**) kinematic chain of a single leg with labeled joint variables; (**c**) Denavit–Hartenberg (DH) frame assignment for the leg; and (**d**) full hexapod with base and platform frames and leg indices.

This section presents a numerical framework for computing the direct and inverse kinematics of a Stewart–Gough platform equipped with preloaded Cardan joints (Figure 3) whose rotational axes do not intersect. Because the joint axes are offset, the classical spherical joint Stewart–Gough model is not applicable; instead, we explicitly model each leg using homogeneous transformation matrices. We first introduce a full Cardan-joint-aware closure constraint and then derive an optimized constraint set that removes rotational redundancy while preserving the solution.

In the previoisly proposed Cardan-joint-aware model [24], the direct kinematics is obtained by solving the full set of 72 scalar equations defined by Equation (6), whereas the optimized Cardan-joint-aware model reduces the number of required equations from 72 to 36 by enforcing only the subset in Equation (14), thereby significantly decreasing the computational load. Both formulations are mathematically equivalent and converge to the same solution for a given set of encoder readings. Therefore, the numerical accuracy is not compared separately, and the analysis focuses solely on differences in computational efficiency. The equivalence of both solutions is discussed in Section 4.7.

### 4.1. Kinematic Description and Coordinate Frames

We define a base frame B attached to the base plate and a platform frame P attached to the moving top plate. The pose of the platform relative to the base is represented by the homogeneous transformation matrix

(5) $$T_{\mathrm{top}}=\left[\begin{array}{cc} R & t\\ 0 & 1 \end{array}\right],\;R\in SO\left(3\right),\;t={\left[x\;y\;z\right]}^{T},$$

where *R* is the top plate rotation matrix and *t* is the translation vector connecting the top and the bottom plate. The orientation can be parameterized, for example, by Euler angles (α,β,γ) such that R=R(α,β,γ).

### 4.2. Full Cardan-Joint-Aware Closure Constraints

For each leg i∈{1,…,6}, we define two transformation chains:

- Platform-side chain: $T_{\mathrm{top}}\;T_{\mathrm{tp},i}$, where $T_{\mathrm{tp},i}$ is a constant transform from the platform frame P to the platform-side Cardan joint reference frame of leg *i*.
- Base-side chain: $T_{\mathrm{bp},i}\;T_{\mathrm{DH},i}$, where $T_{\mathrm{bp},i}$ is a constant transform from the base frame B to the base-side Cardan joint reference frame and $T_{\mathrm{DH},i}$ is a DH transform describing the internal kinematic chain of leg *i*, including both Cardan joints and the cylindrical joint (containing the measured leg length).

A valid configuration must satisfy the loop-closure constraint

(6) $$T_{\mathrm{bp},i}\;T_{\mathrm{DH},i}\;=\;T_{\mathrm{top}}\;T_{\mathrm{tp},i},\;i=1,\ldots ,6.$$

Equation (6) is a matrix equality that corresponds to 12 scalar equations per leg (9 rotational and 3 translational), i.e., 72 scalar equations for the full mechanism. Because rotations in SO(3) have only three independent degrees of freedom, the rotational part of the full constraint contains redundancy. Next, we introduce an optimized constraint set that removes this redundancy and improves numerical conditioning.

### 4.3. Optimized (Reduced) Constraint Formulation

#### 4.3.1. Relative Transformation

For each leg, define the relative transform

(7) $$C_{i}\;=\;T_{\mathrm{bp},i}^{-1}\;T_{\mathrm{top}}\;T_{\mathrm{tp},i}.$$

In a valid configuration, the relative transform matches the leg DH transform,

(8) $$C_{i}\;=\;T_{\mathrm{DH},i}.$$

Let the rotation and translation components be denoted by

(9) $$C_{i}=\left\{R_{C,i},\;t_{C,i}\right\},\;T_{\mathrm{DH},i}=\left\{R_{\mathrm{DH},i},\;t_{\mathrm{DH},i}\right\}.$$

#### 4.3.2. Translational Constraints

Matching translations yields three scalar constraints per leg:

(10) $$e_{t,i}\;=\;t_{C,i}-t_{\mathrm{DH},i}\;=\;0.$$

#### 4.3.3. Rotational Constraints

To compare rotations, define the relative rotation

(11) $$R_{\mathrm{err},i}\;=\;R_{\mathrm{DH},i}^{T}\;R_{C,i}.$$

If the rotations match, then $R_{\mathrm{err},i}=I$. A minimal three-parameter rotation error vector can be obtained from the skew-symmetric part of $R_{\mathrm{err},i}$:

(12) $$e_{R,i}\;=\;\frac{1}{2}\left[\begin{array}{c} {\left(R_{\mathrm{err},i}\right)}_{32}-{\left(R_{\mathrm{err},i}\right)}_{23}\\ {\left(R_{\mathrm{err},i}\right)}_{13}-{\left(R_{\mathrm{err},i}\right)}_{31}\\ {\left(R_{\mathrm{err},i}\right)}_{21}-{\left(R_{\mathrm{err},i}\right)}_{12} \end{array}\right]\;=\;0.$$

This representation provides three independent rotational constraints (locally equivalent to imposing $R_{C,i}=R_{\mathrm{DH},i}$) and eliminates algebraic redundancy from the nine scalar rotation matrix equalities.

#### 4.3.4. Stacked Constraint Vector

For each leg, stack rotational and translational errors into a six-dimensional vector

(13) $$f_{i}\;=\;\left[\begin{array}{c} e_{R,i}\\ e_{t,i} \end{array}\right]\;=\;0,$$

and define the full constraint vector as

(14) $$f\left(\;\cdot \;\right)\;=\;\left[\begin{array}{c} f_{1}\left(\;\cdot \;\right)\\ \vdots \\ f_{6}\left(\;\cdot \;\right) \end{array}\right]\;=\;0.$$

The optimized formulation yields 6 scalar constraints per leg and therefore 36 scalar constraints in total. In practice, this reduction improves numerical conditioning and reduces computation time while preserving the converged solution.

### 4.4. Direct Kinematics

In the direct kinematics problem, the measured leg lengths $l_{i}$ are known, while the platform pose and internal joint variables are unknown. Each leg DH transform $T_{\mathrm{DH},i}$ depends on five internal joint variables (Cardan and cylindrical-joint coordinates), denoted

(15) $$\delta_{i},\;\varepsilon_{i},\;\zeta_{i},\;\eta_{i},\;\theta_{i},\;i=1,\ldots ,6.$$

Collect all unknowns into the vector

(16) $$z={\left[x,\;y,\;z,\;\alpha ,\;\beta ,\;\gamma ,\;\delta_{1},\;\varepsilon_{1},\;\zeta_{1},\;\eta_{1},\;\theta_{1},\;\ldots ,\;\delta_{6},\;\varepsilon_{6},\;\zeta_{6},\;\eta_{6},\;\theta_{6}\right]}^{T}.$$

The system f(z)=0 in Equation (14) is solved iteratively using a Newton–Raphson update

(17) $$z_{k+1}\;=\;z_{k}\;-\;J^{+}\left(z_{k}\right)\;f\left(z_{k}\right),$$

where J(z)=∂f(z)/∂z is the Jacobian and $J^{+}$ denotes the Moore–Penrose pseudoinverse. The pseudoinverse improves robustness when J is ill-conditioned or locally rank-deficient. The iteration stops when

(18) $$∥z_{k+1}-z_{k}∥<\varepsilon_{\mathrm{goal}},$$

with the initial guess $z_{0}$ taken from the previously known configuration.

### 4.5. Inverse Kinematics

In inverse kinematics, the platform pose is prescribed and the unknowns are the leg lengths and internal joint variables. We collect the unknowns as

(19) $$y={\left[l_{1},\;\delta_{1},\;\varepsilon_{1},\;\zeta_{1},\;\eta_{1},\;\theta_{1},\;\ldots ,\;l_{6},\;\delta_{6},\;\varepsilon_{6},\;\zeta_{6},\;\eta_{6},\;\theta_{6}\right]}^{T}.$$

The constraint equations remain

(20) f(y)=0,

and the solution is obtained via

(21) $$y_{k+1}\;=\;y_{k}\;-\;J^{+}\left(y_{k}\right)\;f\left(y_{k}\right),$$

initialized from the most recently known configuration.

### 4.6. Generality of the Formulation

A key feature of the proposed approach is that it is not restricted to a fixed choice of unknown variables. Any subset of variables appearing in the closure relation Equation (6) can be selected as optimization variables, enabling direct, inverse, or hybrid kinematic computations within a unified framework. This generality is particularly useful for mechanisms with non-standard joint geometries, such as the preloaded Cardan joints considered here, where classical closed-form Stewart–Gough expressions are not directly applicable.

### 4.7. Justification of Rotational Constraint Reduction

In the full Cardan-joint-aware formulation, rotational consistency for each leg is enforced by equating the rotation matrices $R_{C,i}$ and $R_{DH,i}$, which yields nine scalar equations per leg. However, these equations are not independent. Rotation matrices are elements of the special orthogonal group SO(3), which is a three-dimensional Lie group. The orthogonality condition

(22) $$R^{T}R=I$$

together with the unit-determinant constraint

(23) det(R)=1

imposes six algebraic constraints on the nine matrix entries, leaving only three independent degrees of freedom. Consequently, enforcing equality between two rotation matrices requires only three independent scalar constraints, and the remaining six equations are redundant.

To obtain a minimal and non-redundant representation of rotational consistency, the optimized formulation introduces the relative rotation

(24) $$R_{\mathrm{err},i}=R_{DH,i}^{T}R_{C,i}.$$

The condition $R_{C,i}=R_{DH,i}$ is satisfied if and only if $R_{\mathrm{err},i}=I$. Instead of enforcing this condition through nine scalar equations, rotational mismatch is characterized by the skew-symmetric part of $R_{\mathrm{err},i}$, yielding the rotational error vector Equation (12). This vector provides a local minimal parameterization of the rotation error in the Lie algebra SO(3). In a neighborhood of the identity, $e_{R,i}=0$ if and only if $R_{\mathrm{err},i}=I$, establishing the equivalence between the rotational error vector formulation and direct rotation matrix equality.

From a numerical standpoint, replacing the overconstrained set of nine scalar equations with a minimal three-dimensional representation significantly improves the conditioning of the resulting nonlinear system. In the full formulation defined by Equation (6), the resulting error equation contains linearly dependent rows associated with redundant rotational constraints, which may lead to ill-conditioning or local rank deficiency during the Newton–Raphson iteration. In contrast, the optimized error formulation defined by Equation (14) yields a square Jacobian matrix whose rows correspond to independent translational and rotational constraints, resulting in consistently lower condition numbers across the workspace.

To further enhance numerical robustness, the Newton updates are computed using the Moore–Penrose pseudoinverse of the Jacobian. This approach provides a well-defined least-squares correction even in the presence of mild ill-conditioning and ensures stable convergence near singular or near-singular configurations. Consequently, the optimized formulation preserves the full kinematic fidelity of the original model while achieving improved numerical stability and reduced computational complexity.

Additionally, the difference between the full Cardan-joint-aware approach and the optimized one is in the improved numerical stability, evaluated by comparing the Jacobian conditioning number defined as

(25) $$\kappa \left(J\right)=\frac{\sigma_{max}\left(J\right)}{\sigma_{min}\left(J\right)},$$

where $\sigma_{max}\left(J\right)$ and $\sigma_{min}\left(J\right)$ are the maximal and minimal Jacobian singular values, respectively. The analyses of comparing conditional numbers for the Cardan-joint-aware and the optimized Cardan-joint-aware approach are presented in Section 5.4.

## 5. Experimental Validation

### 5.1. Experimental Setup

The experimental validation was conducted on a passive hexapod equipped with six LinACE^™^ (https://www.rls.si/eng/linace-absolute-linear-shaft-encoder, accessed on 14 January 2026) absolute linear encoders embedded in the mechanism’s legs. To generate reproducible and spatially diverse test poses, a UR10e collaborative robot (Universal Robots, Novi, MI, USA) [25] was rigidly mounted to the same support frame as the hexapod. A custom top plate with a tool-changer interface was added to the hexapod, enabling direct mechanical coupling to the UR10e end effector. This arrangement ensured a stable connection while allowing the robot to displace the platform into random poses throughout its workspace.

To obtain independent ground-truth pose measurements, a PrimeX 22 OptiTrack motion capture system was used [26]. Six infrared cameras were arranged to cover a compact measurement volume of approximately $0.5\;m^{3}$, maximizing triangulation accuracy within the region of interest. A rigid marker cluster was mounted on the hexapod’s top plate and tracked at full frame rate. Prior to experimentation, the OptiTrack system was calibrated using the standard wand-calibration procedure, followed by rigid-body alignment between the OptiTrack reference frame and the hexapod base frame. The resulting setup provided sub-millimeter positional accuracy and sub-degree rotational accuracy.

During validation, the UR10e robot moved the top plate of the hexapod to a sequence of randomly generated poses within the workspace. At each pose, three data sources were collected synchronously: the 6-DoF ground-truth pose from OptiTrack, the six leg-extension values from the LinACE encoders, and the robot TCP pose, used solely for motion execution and not for accuracy evaluation. A total of 3000 pose configurations were recorded.

### 5.2. Accuracy Evaluation

Two numerical kinematic models were evaluated: the proposed optimized Cardan-joint-aware model that accounts for the specific preloaded Cardan joint geometry and the standard Stewart–Gough formulation based on the spherical joint assumption [7,8]. The non-optimized Cardan-joint-aware model was not included in this comparison, as it yields numerically identical pose estimates to the optimized formulation, differing only in computational efficiency. Both models used the same encoder-derived leg lengths to compute platform pose estimates for comparison against the OptiTrack measurements. Random target configurations were generated by sampling translations within ±50mm of the workspace center and Euler-angle rotations within $\pm 25^{\circ }$. Each candidate pose was validated using inverse kinematics; poses requiring leg lengths outside the mechanical limits were discarded and regenerated.

For each feasible pose, the UR10e robot—attached to the hexapod top plate via the tool-changer interface—manipulated the platform into the corresponding spatial configuration. Once the target pose was reached, the hexapod brakes were engaged to lock the structure. The robot then retracted to avoid occluding the OptiTrack cameras, and all measurements were recorded after a short stabilization interval.

For each of the 3000 valid poses, the encoder readings were processed using both the optimized Cardan-joint-aware and standard Stewart–Gough models. The resulting pose estimates were stored along with the corresponding OptiTrack reference pose for subsequent accuracy evaluation.

Ground-truth and model-estimated poses were compared using separate metrics for translation and rotation. The translational error was computed as

(26) $$\Delta p=∥p_{g}-p_{cm}∥,$$

where $p_{g}$ is the OptiTrack position measurement and $p_{cm}$ is the position estimated by the computational model.

Orientation error was computed using the norm of the rotation-vector representation:

(27) $$\Delta \theta =∥\mathrm{Log}\;\left(R_{g}^{T}R_{cm}\right)∥,$$

where $R_{g}$ is the OptiTrack rotation matrix, $R_{cm}$ is the estimated rotation, and Log(·) denotes the matrix logarithm mapping SO(3) to its rotation-vector representation [23]. This formulation is equivalent to computing the minimal-angle orientation difference.

**Figure 4:**
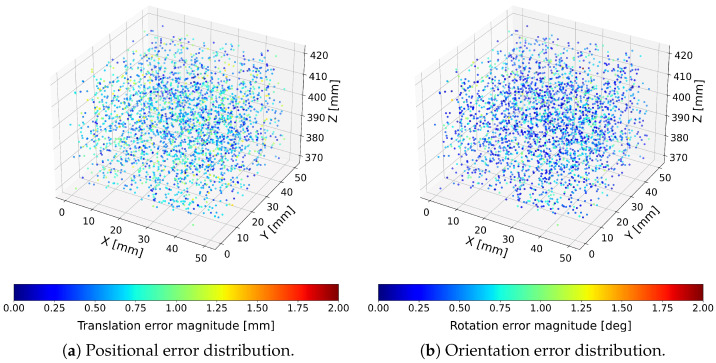
Translation and rotation error distributions obtained using the optimized Cardan-joint-aware model.

**Figure 5:**
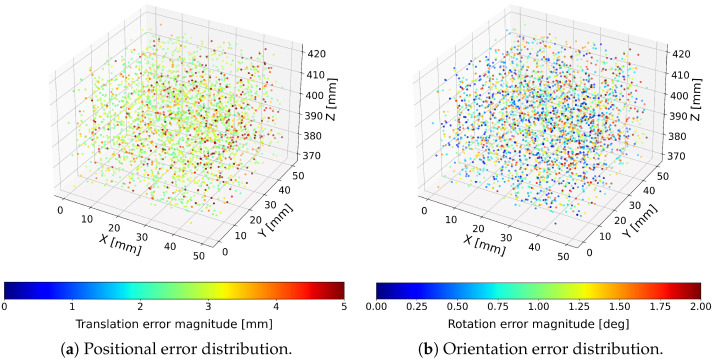
Translation and rotation error distributions obtained using the standard Stewart–Gough model.

To visualize the spatial distribution of errors across the workspace, both translation and orientation errors were represented as color-encoded point clouds, where each point corresponds to a single tested pose and its color encodes the magnitude of the respective error. Positional and rotational errors were visualized separately due to their different physical units. Results for the proposed optimized Cardan-joint-aware and standard Stewart–Gough computational models are presented in Figure 4 and Figure 5.

Across all 3000 tested configurations, the hexapod achieved mean translational errors below 0.62mm and mean orientation errors below $0.26^{\circ }$ when evaluated using the optimized Cardan-joint-aware model. The aforementioned model consistently outperformed the standard Stewart–Gough model [13,14,20]. These results demonstrate that the integration of absolute linear encoders, combined with a joint-geometry-aware kinematic model, enables sub-millimeter and sub-degree pose estimation, confirming the suitability of the sensor-enhanced hexapod for high-precision industrial fixturing applications [9,10]. The corresponding statistical analyses for the optimized Cardan-joint-aware model and standard Stewart–Gough model are summarized in Table 1.

### 5.3. Convergence Evaluation

**Figure 6:**
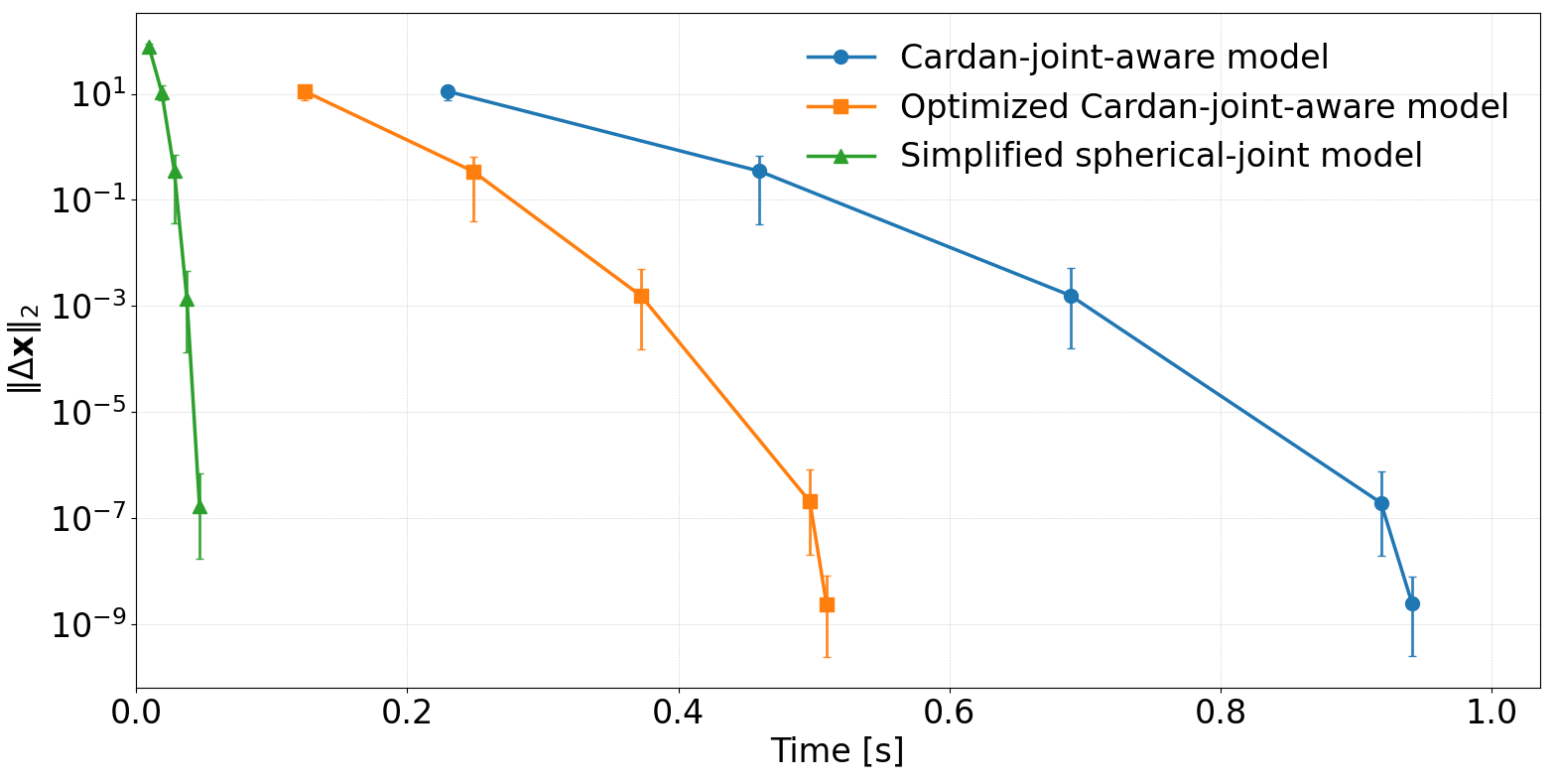
Convergence of different methods.

In addition to the workspace error statistics, we analysed the convergence behavior of the three inverse kinematic solvers by plotting the parameter update norm ${∥\Delta x∥}_{2}$ given in Equation (28) as a function of computation time [23] (see Figure 6).

(28) $${∥\Delta x^{\left(k\right)}∥}_{2}={∥x^{\left(k+1\right)}-x^{\left(k\right)}∥}_{2}=\sqrt{\sum_{i=1}^{n}{\left(x_{i}^{\left(k+1\right)}-x_{i}^{\left(k\right)}\right)}^{2}}.$$

All three methods exhibit a nearly linear decrease on the semi-logarithmic scale, indicating stable and well-conditioned Newton iterations (see Figure 6). The two detailed model variants (the Cardan-joint-aware model, which solves the full equation system in (6), and the optimized Cardan-joint-aware model, which solves the reduced systems (12) and (14)) are both based on the same matematical model and converge to essentially identical final parameter values. Consequently, the translational and rotational errors they produce are indistinguishable within numerical resolution, and the only practical difference between them is the time required to reach the stopping criterion, with the modified formulation being consistently faster. The reported computation times were obtained on a desktop PC equipped with an AMD Ryzen 9 9950X3D 16-core processor (Advanced Micro Devices, Inc., Santa Clara, CA, USA), and all solvers were implemented with symbolic computation in Python 3.11.6.

In contrast, the standard Stewart–Gough model converges much more rapidly in time, with its convergence curve shifted far to the left, but the error distributions in the 3D plots reveal substantially larger translation and rotation errors across the workspace. This indicates that, although numerically efficient, the simplified formulation does not reproduce the true mechanism kinematics as accurately as the proposed models. It is therefore best regarded as a fast approximation or an initial-guess generator rather than a replacement for the full kinematic description when high absolute accuracy is required.

Note that the relatively long computation times are primarily a consequence of the symbolic calculation performed using the SymPy package (version 1.12). While this symbolic formulation offers substantial flexibility, particularly the ability to accommodate arbitrary kinematic structures, as discussed in Section 4.5, it also makes the approach too computationally demanding for real-time execution on the Raspberry Pi. As part of our future work, we plan to derive an optimized set of analytical expressions tailored to a specific hexapod configuration, enabling the kinematic calculations to be executed directly on the Raspberry Pi with significantly reduced computational effort.

### 5.4. Numerical Stability Evaluation

**Figure 7:**
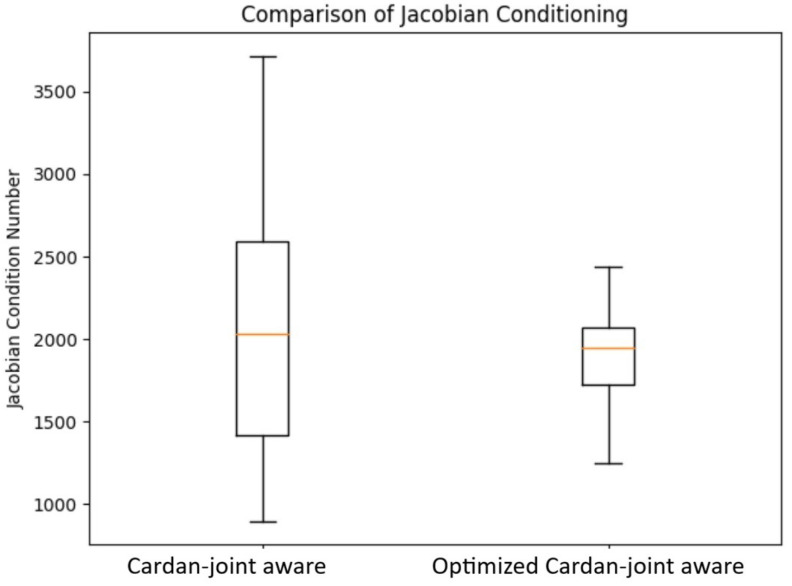
Comparison of the mean and standard deviation for the Cardan-joint aware and optimized Cardan-joint aware models.

To prove the numerical stability benefits of the optimized versus Cardan-joint-aware approach, we calculated the conditioning number (Equation (25)) in each iteration across all 3000 tested configurations for both methods. The results, which clearly outline the improved numerical stability of the optimized Cardan-joint-aware approach, are shown in Figure 7.

### 5.5. Practical Use-Case Evaluation

Finally, we performed a series of tests to demonstrate that the robot can accurately manipulate the hexapod after it has been randomly displaced by a human operator. In this experiment, we used the hexapod equipped with six LinACE™ absolute linear encoders together with a UR10e collaborative robot. Both the robot and the hexapod were fitted with a Destaco QC30–TP30 tool-changer interface (Destaco, Auburn Hills, Michigan, USA), and communication between the two systems was handled through a ROS.

During each trial, the human operator manually displaced the hexapod and then engaged its hydro-mechanical brakes to lock its position. The robot then read the pose of the hexapod’s top plate, approached it, and connected the two halves of the clamping mechanism. Achieving this requires sub-millimeter absolute accuracy.

We performed 50 repetitions of the experiment and achieved a 100 % success rate. Videos of the experiment are provided in the Supplementary Materials.

## 6. Conclusions

This work experimentally validated a direct kinematics formulation for a passive hexapod fixture equipped with absolute linear encoders embedded in its cylindrical leg joints. Using a UR10e robot to move the mechanism and an OptiTrack PrimeX 22 motion capture system as an external reference, 3000 randomly selected poses were recorded and evaluated. Across all tested configurations, the proposed Cardan-joint-aware model achieved mean translational errors below 0.62 mm and mean orientation errors below 0.26°, confirming that the combination of encoder instrumentation and an accurate joint geometry model enables high-precision pose estimation suitable for industrial fixturing and flexible assembly applications.

The aim of this work is not to introduce a new numerical solver, but to address a fundamental modeling limitation that arises when classical Stewart–Gough formulations are applied to mechanisms with non-intersecting Cardan joint axes. The primary contribution is therefore a geometry-consistent kinematic constraint model that explicitly captures the true joint structure and enables reliable pose reconstruction using only internal encoder measurements, without relying on empirical calibration or data-driven correction methods.

The choice of an optical measurement system as the ground-truth reference requires some clarification. This system enabled the acquisition of a large number of measurements within a short time, allowing the entire operational workspace of the hexapod to be thoroughly sampled. Although its absolute accuracy is lower than that of high-precision coordinate metrology equipment, it remains sufficient for evaluating performance in the intended industrial, non-human-interaction applications, as well as for robot-assisted manipulation of the hexapod. Using a precision coordinate measuring device would yield higher absolute accuracy and might demonstrate even better agreement with the hexapod’s built-in sensors; however, such increased precision would not meaningfully affect the assessment of the system’s practical usability.

The comparison between the three inverse kinematics variants showed a clear trade-off between model fidelity and computational cost. The optimized Cardan-joint-aware model is relatively slow, as all kinematic quantities are represented and manipulated symbolically, but this design choice allows the same model to be used consistently for both direct and inverse kinematics and to handle arbitrary input vectors, with the remaining hexapod parameters being inferred automatically from the symbolic equations. Evaluating this unified symbolic formulation in more detail, including its behavior for different choices of input variables, is an important topic for future work.

The two proposed models, which both incorporate the modified Cardan joint geometry, converge to essentially identical pose estimates and therefore yield the same translation and rotation error distributions over the workspace. Their only practical difference lies in runtime: the optimized Cardan-joint-aware model reaches the same convergence threshold significantly faster, making it the preferable choice for real-time or high-throughput implementations. The standard Stewart–Gough model, in contrast, exhibits much smaller computation times and a rapid decay of the Newton update norm, but its translational and rotational errors are substantially larger and show stronger spatial variation. This indicates that the standard Stewart–Gough model does not capture the true mechanism kinematics with sufficient accuracy and is best used as a fast approximation or as an initial-guess generator rather than as a stand-alone solution when absolute accuracy is critical.

Overall, the results demonstrate that a passive hexapod equipped with absolute encoders and driven by a numerically efficient, geometry-aware direct kinematics model can provide reliable 6-DoF pose feedback without resorting to external measurement systems during operation. This makes the concept particularly attractive for reconfigurable fixtures in flexible manufacturing, where rapid changeovers and reproducible positioning are essential. In particular, the ability to reconstruct the platform pose solely from internal encoder measurements is a key enabling technology for human–robot collaboration: any manual repositioning of the hexapod by an operator would otherwise be unobservable and therefore unusable by the robot controller in the absence of such a feedback system. Future work will focus on extending the approach to dynamic loading conditions, studying long-term stability under thermal and mechanical drift, and exploring alternative high-level, task-oriented optimization criteria that enable optimal changeover from one workpiece to another. Moreover, we will derive an optimized set of analytical expressions tailored to a specific hexapod configuration, enabling the kinematic calculations to be executed directly on the Raspberry Pi.

## Figures and Tables

**Table 1:** Statistical summary of translation and rotation errors for the optimized Cardan-joint-aware model compared with the standard Stewart–Gough model. The paired *t*-tests confirm that the proposed model yields significantly lower errors in both translation and rotation. Negative *t*-values indicate the superior performance of the proposed model across all 3000 evaluated configurations.

Metric	Optimized Cardan-Joint-Aware Model	Standard Stewart–Gough Model
Mean translation error μ [mm]	0.6205	3.1684
Std. dev. translation error σ [mm]	0.2609	0.9094
Mean rotation error μ [deg]	0.4657	1.0037
Std. dev. rotation error σ [deg]	0.2417	0.5117
Paired *t*-test (translation)	t=−131.997,p<0.001	
Paired *t*-test (rotation)	t=−43.974,p<0.001	

## Data Availability

Data is contained within the article or Supplementary Materials.

## Supplementary Materials

The following supporting information can be downloaded at https: //www.mdpi.com/article/10.3390/s1010000/s1, Video S1: Hexapod demo.
